# Exploring the spiritual needs of patients receiving palliative care and their caregivers: a qualitative study

**DOI:** 10.1186/s12904-026-02200-2

**Published:** 2026-06-23

**Authors:** Gülay Turgay, Banu Çevik, Sultan Kav, Ebru Akgün Çıtak

**Affiliations:** 1 Başkent University Health Services Vocational School, Ankara, Turkey; 2 Faculty of Health Sciences, Department of Nursing, Başkent University, Ankara, Turkey

**Keywords:** Caregivers, Interviews, Needs assessment, Palliative care, Patients, Qualitative research, Spirituality

## Abstract

**Background:**

Diagnosing chronic or life-threatening illnesses can lead to spiritual struggles for patients and their caregivers. This study aims to explore the spiritual needs of patients receiving palliative care and their caregivers.

**Methods:**

The qualitative study was conducted with patients and their caregivers in the palliative care unit of a foundation hospital in Ankara. Semi-structured interviews were conducted with 22 patients and 22 caregivers. The interview questions for patients focused on health-related issues, challenges, coping strategies, significant aspects of their lives, and any changes in their beliefs during illness. Similarly, caregivers were asked four semi-structured questions regarding their challenges, coping strategies, the most meaningful aspects of their lives, and any changes in their beliefs as caregivers. Data analysis was performed using Colaizzi’s phenomenological approach.

**Results:**

The most common diagnoses among patients were end-stage renal failure, chronic liver disease, lung cancer, and ovarian cancer, respectively. Data analysis revealed four main themes and eight sub-themes. The main themes identified were: “Functional Disability,” “Transcendence and Family Support,” “Acceptance,” and “Psychological Pain.” Four main themes and three sub-themes emerged from the caregiver data. The main themes were: “Care Burden,” “Lack of Information,” “God/Higher Power,” and “Glory of Care.” The “Care Burden” theme encompassed three sub-themes: “Physical Problems,” “Stress,” and “Economic Problems.”

**Conclusions:**

The results of this qualitative study indicate that spiritual values are indispensable for the patients and caregivers, and they perceive support from God, family, and friends even during the most difficult times.

## Background

Palliative care is an approach aimed at improving the quality of life for individuals with life-threatening diseases and their families [1]. It involves the early diagnosis and management of physical, psychosocial, and other issues that arise in individuals, enabling them cope with pain and addressing a range of concerns beyond physical symptoms [2, 3]. The aging global population and the increasing prevalence of chronic diseases have heightened the demand for palliative care services worldwide. According to the World Health Organization (WHO), 78% of adults in low- and middle-income countries require palliative care, yet only 14% are estimated to receive it. As such, it is critical for countries to assess and address the palliative care needs of their populations to strengthen available services [3]. Chronic or life-threatening illnesses not only affect patients but also have a significant impact on their caregivers and families [4]. Consequently, providing patient- and family-centered care in palliative settings is essential. Evidence suggests that patient- and family-centered interventions can reduce caregiver distress, alleviate perceptions of unmet need, prepare family members for caregiving, and improve bereavement outcomes [5].

A diagnosis of a life-threatening illness can lead to not only physical but also spiritual struggles for both the individual and their caregivers. This spiritual journey may foster growth and transformation for some, while for others, it may lead to heightened anxiety, depression, stress, and spiritual distress, or a combination of both. Recent evidence indicates that spiritual health–based interventions can significantly improve psychological indicators such as anxiety, hope, and quality of life, underscoring the importance of spirituality in patients’ overall well-being during such challenging times [6]. Physical problems that arise in the individual can affect other areas; emotional-spiritual effects can cause changes in the physical field [7, 8]. For this reason, when evaluating the individual, it should be handled with a holistic approach with physical, psychosocial, emotional, cultural, and spiritual dimensions [9–11].

Holistic health of an individual is possible through the realization of all care practices, including spiritual care. Spiritual care specifically includes initiatives supporting individuals during times of distress, sadness, or stress resulting from life crises, when they experience profound feelings of loneliness and hopelessness, guiding them to perform their customs, worship, and rituals, while helping them rediscover meaning and purpose in their lives. Spiritual care is now recognized as a fundamental component of palliative care [9–11]. Research further indicates that individuals’ spiritual, religious, and cultural beliefs significantly influence health decisions and outcomes, including coping mechanisms, quality of life, and pain management [12–13].

Therefore, health professionals should adopt holistic care when gathering data for treatment and care, and should obtain information on social, psychological, and spiritual needs, alongside physical problems [14]. Spiritual needs are basic for individuals, as they are the needs that fulfill the spiritual deprivation of the individual and increase their spiritual strength. These needs become more pronounced when faced with emotional stress, physical illness, or life-threatening conditions [15]. The basic spiritual needs for individuals are defined as trust, hope, love, truth, the desire to find meaning and purpose in life, forgiveness, experience, sensuality, speech, consolation, rituals, prayer and worship. The individual fulfills these needs through human relationships (with others) or by establishing a connection with the Creator or Great Power. In conducting a spiritual assessment, it is essentials to first identify factors specific to the individual, their spiritual or religious affiliations, beliefs, practices, and experiences; sources of strength and support; concerns related to meaning and suffering; cultural norms and preferences; as well as their hopes, values and fears [16, 17]. It has been emphasized in the research that the individual can contribute to care in this direction by determining their spiritual needs [11, 18]. All health professionals can perform spiritual assessment to provide spiritual care. However, nurses are uniquely positioned to provide spiritual care as they are the only healthcare professionals who are with the patient 24/7. The literature highlights that trust-based relationships established by nurses with patients and their families are crucial in effectively identifying spiritual needs. Furthermore, spiritual care has been shown to increase patient and family satisfaction and improve overall quality of life [18, 19].

Recent studies in Turkey have begun to explore the spiritual dimensions of palliative care from the perspectives of family caregivers and healthcare providers. For example, Uzun et al. (2024) investigated the spiritual needs of family caregivers through interviews conducted solely with caregivers, while Kurtgöz and Edis (2023) examined spiritual care from the perspectives of both caregivers and nurses, primarily emphasizing how care is provided and the institutional or provider-related barriers encountered [20, 21]. Although these studies contribute valuable insights, important gaps remain. First, the voices of patients themselves have not been explored alongside those of their family caregivers, limiting the ability to compare and integrate perspectives across these two closely interconnected groups. Second, prior research has largely focused on identifying needs or describing barriers, but less attention has been paid to how spiritual care can be integrated holistically into palliative care practice at the patient, family, and institutional levels. Furthermore, previous work has concentrated primarily on cancer patients and relatively early illness trajectories, leaving unexplored the ways in which spiritual needs may evolve across diverse diagnoses and over longer periods of care. Addressing these gaps is crucial for advancing a more comprehensive and culturally sensitive understanding of spirituality in palliative care. Therefore, this qualitative study was conducted to explore the spiritual needs of individuals receiving palliative care and their family caregivers, with particular attention to how these needs are experienced, compared, and contextualized within the caregiving relationship.

### Research questions

1. What are the spiritual needs of individuals receiving palliative care and their caregivers?
2. What individuals receiving palliative care and their caregivers do in the spiritual dimension to cope?

## Methods

This study employed a descriptive and qualitative design, which is appropriate for exploring human experiences in depth [22, 23]. Purposive sampling was utilized to recruit patients receiving palliative care and their primary caregivers. Data collection was conducted in two phases due to interruptions caused by the COVID-19 pandemic. Six patient and their caregivers’ interviews were conducted prior to the onset of the pandemic, beginning in October 2019, and resumed following the easing of restrictions, continuing until March 2022. Data were collected through in-depth, semi-structured interviews conducted face-to-face within the palliative care unit of Foundation University Hospital, each lasting approximately 40 to 60 min. Before participation, all individuals received comprehensive information regarding the study and were informed that the interviews would be audio-recorded for qualitative analysis purposes. Written informed consent was obtained from all participants. To maintain confidentiality, all personal identifiers were removed, and participants were anonymized. Written documentation generated during data collection was not disclosed to any third parties outside the research team. Audio recordings were securely stored in an encrypted file on the principal researcher’s personal computer, with access restricted to authorized members of the research team only.

### Setting

The study was conducted in the palliative care center of a foundation university hospital in Ankara, Türkiye.

### Participants

The population of the study consisted of individuals receiving treatment in the Palliative Care Unit of a foundation university and their caregivers. Individuals who agreed to be interviewed constituted the sample and in-depth interviews were conducted. Interviews were continued until the responses from the interviews were repeated and data saturation was reached. The study was completed with 22 patients and 22 caregivers.

### Data collection

“Data were collected using two instruments: the Personal Information Form and the Semi-Structured Questionnaire. The Personal Information Form captures sociodemographic characteristics, including age, gender, educational status, marital status, occupational status, and income level, as well as the individual’s disease experience. The Semi-Structured Questionnaire was designed separately for individuals receiving palliative care (Table 1) and their caregivers (Table 2).

**Table 1:** Questions for the individual receiving palliative care

1. What difficulties have you had with your health?
2. What are the spiritual values that help you cope in difficult times?
3. What are the things you value most in your life?
4. What changes have occurred in your faith after the illness?

**Table 2:** Questions for caregivers

1. What are the situations where you have difficulties related to the health of the person you care for?
2. What are your spiritual values that help you cope with difficult times related to the person you care for or yourself?
3. What are the things you value most in your life?
4. What kind of changes have occurred in your faith after you started caring for your patient?

### Data analysis

Data were analysed using Colaizzi’s (1978) phenomenological method [24] The transcripts of all interviews were read several times to ensure familiarisation and gain a holistic understanding of the participants’ experiences. Significant statements were then identified, and their underlying meanings were interpreted using bracketing, where researchers set aside their own assumptions to remain close to participants’ perspectives. The meanings were subsequently clustered into themes and sub-themes that captured the essence of patients’ and caregivers’ spiritual, psychological, and physical experiences. A comprehensive description was then developed and refined into a fundamental structure representing the phenomenon. Finally, this structure was shared with participants for validation, and their feedback was incorporated to strengthen the credibility and trustworthiness of the findings.

### Study rigour

Four key criteria are used to ensure the rigor of qualitative research: credibility, reliability, confirmability, and transferability [25]. These criteria were carefully considered throughout the research process. The study involved four researchers who conducted face-to-face interviews, each lasting 30 min. To enhance credibility, participants reviewed the identified themes and evaluated the researchers’ interpretations. Reliability was strengthened through researcher triangulation, with multiple researchers engaged in both data collection and analysis. Confirmability was ensured by integrating direct quotations from participants into the findings. Additionally, participants provided feedback on the extent to which the identified themes accurately reflected their experiences. Transferability was supported by offering detailed information on the sampling method, participant characteristics, and interview setting.

The final findings were generated after all researchers agreed that the edited content most accurately reflected the participants’ spiritual needs. The GRAMMS checklist was used for reporting [26].

## Results

Among the participants, 59.1% of the patients were female, with a mean age of 69.81 ± 11.89 years (range: 47–90). When grouped, 1 patient (4.5%) was between 40 and 49 years, 4 patients (18.2%) were between 50 and 59 years, 5 patients (22.7%) were between 60 and 69 years, 7 patients (31.8%) were between 70 and 79 years, and 5 patients (22.7%) were aged 80 years and above. It was found that 31.8% of the patients had completed primary education, and 59.1% were married. Additionally, 40.9% of the patients lived with their spouses. The mean duration since diagnosis was 9.18 ± 2.08 years (range: 3–16). The most common primary diagnoses were end-stage renal failure (36.4%), chronic liver disease (18.2%), lung cancer (13.6%), and ovarian cancer (13.6%).

Among the caregivers, 72.7% were female, with a mean age of 54.77 ± 13.71 years (range: 29–70). When grouped, 1 caregiver (4.5%) was between 20 and 29 years, 4 caregivers (18.2%) were between 30 and 39 years, 3 caregivers (13.6%) were between 40 and 49 years, 3 caregivers (13.6%) were between 50 and 59 years, 10 caregivers (45.5%) were between 60 and 69 years, and 1 caregiver (4.5%) was aged 70 years and above. It was found that 36.4% had a university-level education, and 81.8% were married. Additionally, 68.2% of the caregivers were not employed. Regarding health conditions, 31.8% had hypertension, 22.7% had diabetes, and 4.5% had heart disease. In terms of their relationship to the patient, 36.4% were daughters, 27.7% were sons, 27.3% were spouses, and 13.6% were other relatives (siblings or mothers). The mean duration of caregiving was 3.36 ± 1.52 years (range: 1–6).

In this study, which aimed to identify the spiritual needs of individuals receiving palliative care, five main themes and eight subcategories emerged from the patients’ perspectives. The main themes identified were: ‘Functional Disability,’ ‘Transcendence,’ ‘Family Support,’ ‘Acceptance,’ and ‘Psychological Suffering.’ The theme of Functional Disability included two subcategories: ‘Pain’ and ‘Being Dependent.’ The theme of Transcendence encompassed the subcategory of ‘Spiritual Empowerment.’ The theme of Family Support included the subcategory of ‘Social Isolation.’ Lastly, the theme of Acceptance comprised three subcategories: ‘Embracing the Situation,’ ‘Rebalancing,’ and ‘Finding Peace’ (Table 3).

**Table 3:** Categories related to the statements of individuals receiving palliative care

Themes and sub-categories
1. Functional Disability Pain Dependency
2. Transcendence Spiritual empowerment
3. Family support Social Isolation
4. Acceptance Adopting the Situation Rebalancing Finding Peace
5. Psychological Suffering

### Themes and sub-categories for patients

The themes and subthemes are presented below;

#### Theme 1: Functional disability

All participants reported experiencing functional disabilities. Ten participants stated that they were no longer able to perform activities they had previously managed, such as bathing, grocery shopping, cooking, and caring for their grandchildren. Some statements related to this theme are presented below.

“Now I find it difficult or even impossible to do simple household chores, which makes me very sad.” (P4).

“I can’t help my loved ones because I can’t do anything, I promised to take care of my grandchildren, but I can’t even take care of myself anymore.”(P6).

“I used to go for a walk every day….but now even taking a step inside the house is tiring….”(P14).

“It saddens me that I can’t wear my own clothes, I can’t even tie my shoes.”(P17).

Regarding the pain subcategory of functional disability, patients reported that pain physically exhausted them (n = 5), persisted without relief (n = 3), led to feelings of helplessness (n = 3), and was unbearable (n = 6). Statements reflecting these experiences are presented below.“

I cannot sleep during the night because of my pain, I am always tired during the day and I sleep all the time” (P14).

“I want to be strong but it is getting harder and harder to fight these pains.” (P5).

“The pain is like a prisoner of my body, I can’t feel free.” (P18)

“Sometimes it is so unbearable that I feel like I have completely lost the taste of life.“(P1).

“Waking up with the same pains every day becomes unbearable and I don’t know when they will go away or how severe they will get.” (P22).

The other sub-categories of functional disability is “being dependent”. Two of the statements of seven patients related to this theme are included.

“My children take good care of me, my daughters are always with me. But what makes me sad is that I am always waiting for help from them while everyone else is doing their own work.” (P7).

“I don’t know what I would do without my husband, he does everything.” (P10).

#### Theme 2: Transcendence

Six participants stated that their physical disabilities prompted them to seek a deeper meaning in life. Regarding the subcategory of spiritual empowerment, they reported that their illness led them to change their perspectives on life, become more patient, find joy in small things, draw strength from prayer, and experience an increase in hope. Some patient expressions related to this theme are given below.

“With my illness, even the easy things became difficult….But then these difficulties made me a stronger person.” (P11).

“…. Rediscovering myself has been good for me in this process. First of all, I learned to believe in myself. I pray more than I have ever prayed, I cry more than I have ever cried.” (P9).

“My spiritual belief and prayers keep me going” (P4).

“This process, this illness has taught me how precious life is.” (P2).

#### Theme 3: Family support

Nearly all participants reported feeling supported by their families. However, within the subtheme of social support, they noted a significant decline in their connections with friends. Some of the patient statements related to this are presented.

“We used to drink tea and chat with friends and social contacts; we used to have a good time together. Now no one comes by, it’s as if everyone is slowly cutting me out of their lives. …”(P6).

“I used to see my friends often and spend time outside, but now no one calls me anymore. I feel lonely. Even though my family is with me, being cut off from my circle of friends makes me feel excluded.” (P13).

#### Theme 4: Acceptance

Participants reported experiencing significant life changes following their diagnosis. Their adaptation to the illness varied, with different approaches observed throughout the process. This adjustment was structured around three subthemes: ‘Adopting the Situation,’ ‘Rebalancing,’ and ‘Finding Peace.’ Some participants initially struggled with their diagnosis but later came to accept it as part of their lives. They described a process in which they first sought to understand their illness, then focused on what they could still do despite the challenges, and eventually learned to seek help when needed. Others noted that acceptance made coping easier, leading them to a sense of inner peace. They attributed this peace to factors such as spiritual support, the presence of family members, and the ability to find meaning in life.

“At first it was very difficult to accept my illness. I felt lost…” (P5)

“…I learned to get help, which was very important. Now, when I accepted the disease, things became easier…” (P13).

“…When one accepts life as it is, one somehow reaches peace…” (P16).

#### Theme 5: Psychological suffering

Five participants reported experiencing psychological pain that was more profound than physical pain. They described an internal emptiness that manifested as persistent suffering, which they found more challenging to cope with than physical pain. Statements reflecting this theme are presented below.“

“Loneliness is one of the hardest parts of dealing with this disease. No one can see the pain inside me.”(P12).

“I realized that mentally everything is much more difficult. I can ease the physical pain a little bit, but the mental pain is something else.” (P8).

“Sometimes it’s impossible to describe how deep the pain is….”(P15).

Four main themes and three sub-categories were identified regarding the caregivers of patients in palliative care centers. The main themes are “Burden of Care”, “Lack of Knowledge”, “God/Godly Power” and “The Greatness of Care”. The care burden theme has three sub-categories: “Physical Problems”, “Stress” and “Economic Problems” (Table 4). Caregivers are coded as G.

**Table 4:** Categories related to the statements of caregivers of individuals receiving palliative care

Themes and sub-categories
1.Burden of care Physical Problems Stress Economic Problems
2. Lack of knowledge
3. God/Supreme Power
4. The Sublimity of Care

#### Theme 1: Burden of care

Palliative care presents significant challenges not only for patients but also for their caregivers. Most participants reported experiencing physical health issues while providing care, and eight caregivers expressed difficulty balancing work and caregiving responsibilities, leading to economic hardship. Additionally, four caregivers stated that witnessing a loved one’s dependence on others for daily needs was emotionally exhausting, heightening their anxiety and stress about the future. Statements reflecting these experiences are presented below.“

“My own health has deteriorated while caring for my mother. I am physically exhausted and find it difficult to continue for long periods of time. I have also had to take unpaid leave from my workplace, which puts a strain on me financially.” (G2).

“The extra expenses that come with illness put us in debt.” (G4)

“My mother, who took care of everything for us until now, can’t do anything without us anymore. It’s really hard, it’s emotionally devastating, every day my anxiety and stress increases exponentially.” (G11).

“At my age, it’s very difficult to stay awake all night long, to put my husband down, to lift him up, I ache everywhere.”(G16).

#### Theme 2: Lack of knowledge

Lack of knowledge is a significant challenge faced by caregivers. Insufficient understanding of palliative care not only hinders their ability to adequately meet patients’ needs but also increases their emotional and psychological burden. Sixteen participants reported a lack of knowledge about the disease and its treatment, which made decision-making processes difficult. They expressed concerns about having no one to consult regarding the challenges they encountered, relying instead on internet searches for information, and feeling anxious about the possibility of unintentionally harming their loved ones. Statements reflecting these concerns are presented below.

“I don’t have enough knowledge about the disease and treatment, so I find it very difficult to make decisions, I don’t know whether I have made the right decision or not. When I have difficulties, I can’t consult anyone, I just search for solutions on the internet.” (G5).

“Because of the lack of knowledge, I am constantly worried while caring for my patient. I think a lot about whether I’m doing something wrong, whether I’m harming her. It’s really hard not to be able to consult anyone to make a good decision.” (G7).

“Sometimes I have questions about my patient’s treatment. This process is very stressful because I always question whether I am doing the right thing.” (G10).

#### Theme 3: God / Supreme power

Caregivers faced significant challenges throughout the caregiving process. Twelve caregivers stated that their ability to remain patient while fulfilling this demanding role was primarily derived from the strength they received from God. Additionally, two caregivers mentioned that drinking coffee played a crucial role in their coping mechanism, as it provided them with a moment of solitude and re-motivation during this process.“.

“I pray to God, because sometimes I feel like I cannot carry this burden on my own.” (G5).

“Without God’s help, I find it very difficult to take care of my patient.” (G13).

“I thank God every day because God gives me patience and strength.” (G4)

“Sometimes I get so overwhelmed here and I just want to be by myself, just me and my cup of coffee. A cafe for coffee would be great. Then I recharge, I get stronger again, I go back to my mom happier…” (G8).

#### Theme 4: The sublimity of caregiving

While most of the caregivers stated that caregiving is difficult, almost half of them stated that caregiving makes people happy and gives them peace of mind; three of the caregivers stated that they see it as an opportunity for them and that God has bestowed this reward on them.

The sentences containing these statements are given below.

“Providing care can be very demanding, but it is enough for my mother to say ‘God bless you’.” (G5).

“While caring for my patient, I also find my own inner peace, it makes me happy to be of use to her.” (G1).

“When I think about what I can do to make my brother’s life more meaningful, I see it as a greatness.” (G14).

“Being there for him in his time of need gives me a different value every day.” (G19).

## Discussion

Identifying the spiritual needs of individuals receiving palliative care and their caregivers, understanding the challenges they face in relation to these needs, and developing strategies to address these challenges are of great importance in terms of improving palliative care processes.

The findings of this study provide valuable guidance for health professionals to explore and understand the spiritual needs of patients and caregivers at an early stage and to plan tailored interventions. Palliative care patients often require prolonged clinical monitoring and must cope with multiple and fluctuating symptoms [3, 16, 19]. Therefore, addressing not only the patients’ but also the caregivers’ spiritual, emotional, and informational needs is an essential component of the care process [20, 21]. Recent evidence further shows that spiritual care interventions in palliative care enhance quality of life and coping skills that spiritual care competence of health professionals plays a critical role in care quality and that spiritual support reduces caregiver burden and distress [9, 10, 27, 28].

Caregivers often spend long periods providing continuous care while also managing daily responsibilities, which exposes them to complex emotions ranging from love and loyalty to regret and sadness. This demanding role requires not only physical endurance but also strong psychological and spiritual resilience [7]. Recent studies emphasise that spirituality supports caregivers in finding meaning, strengthening resilience, and coping with caregiving challenges [8, 20, 28, 29]. Moreover, spiritual strength empowers individuals to recognize their potential, build self-confidence, foster love and forgiveness, and cope with suffering [4, 30]. Current findings indicate that caregivers of palliative care patients encounter a range of challenges, including sadness, exhaustion, stress, helplessness, anger, depression, problems in work-life, difficulties in caregiving, fatigue, challenges in caring for their own families, and insomnia [16, 20, 21, 31]. This study focused on the spiritual needs of caregivers and important themes and subthemes were obtained through one-on-one interviews. Spiritual needs of caregivers included the themes of “Care Burden,” “Information Deficit,” “God/Higher Power,” and “The Glory of Care.” Analysis of these themes revealed that while caregivers experience physical fatigue due to the burden of care, they also experience spiritual peace which arises from the comfort and sense of fulfillment gained from meeting the needs of their loved ones. A study by Öner (2023) found that caregivers of oncology patients perceived caregiving as a moderate burden, with the burden increasing as social support decreased [32]. Caregivers strive to balance their own needs with the needs of the individual they care for. Social support functions as a crucial mechanism, sharing caregiving responsibilities, offering material and moral support, and equipping the caregiver with the knowledge and skills needed to cope with stressful situations. The social support perceived by caregivers can mitigate the difficulties they encounter and help them maintain their individuality. Conversely, a lack of social support can lead to problems in physical, psychological, and social dimensions for caregivers [33, 34]. One study highlighted that caregivers’ family relationships were disrupted, and their family responsibilities negatively affected due to their caregiving roles, leading to psychological distress [33]. Vespa et al. (2018) found that caregivers with low spiritual well-being experienced lower quality of life, self-rejection, and difficulties with emotional regulation [35]. Öksüz et al. (2013) reported increases in psychological symptoms, including depression, anxiety, anger, and mental distress among caregivers [36]. Additionally, Yıldız et al. (2016) found that caregivers primarily relied on “hoping” as a coping strategy and turned to spiritual practices to manage heightened stress levels [33]. The themes identified in these studies align with the findings of our research, emphasizing the importance of addressing the multifaceted needs of caregivers. These results underscore the necessity of considering caregivers from a holistic perspective and ensuring that their diverse needs are met.

In our study, the themes reflecting patients’ perspectives on their spiritual needs were identified as “Functional Disability,” “Transcendence,” “Family Support,” “Acceptance,” and “Psychological Pain.” Addressing patients’ spiritual needs can facilitate acceptance of illness and encourage future planning, which in turn positively influences the healing process and enhances hope. Recent evidence shows that caregivers who engage in spiritual coping report better psychological adjustment and less anxiety and depression [13]. Similarly, outpatients with cancer’s spiritual beliefs are significantly associated with higher resilience during treatment [12]. Interventions promoting spiritual coping among family caregivers have demonstrated improvements in coping ability and wellbeing [37]. Patients with strong spiritual values are also able to draw upon their beliefs to cope with illness-related challenges such as pain, uncertainty, and life stressors, thereby supporting psychological adjustment and recovery.

During the course of their illness, individuals benefit not only from medical interventions but also from their spirituality and faith in managing life-threatening situations [16, 38]. Identifying and addressing the spiritual needs that arise during critical moments in an individual’s life can be achieved through personalized spiritual care provided by nurses. In a study conducted by Liu et al. (2023), 103 patients with chronic heart failure were examined, with a comparison made between patients receiving palliative care (*n* = 54) and those receiving standard care (*n* = 49). The findings indicated that palliative care practices significantly improved patients’ physical and mental conditions, alleviated negative emotions, and contributed to the development of effective coping mechanisms. Furthermore, it was concluded that this approach enhances patient satisfaction and improves quality of life [19].

When patients’ spiritual needs are unmet, they often experience spiritual distress. In this study, nearly half of the patients reported suffering from feelings of loneliness and mental fatigue (such as worry, fear, and anxiety) rather than physical pain, with the emotional pain being so profound that it was difficult to describe. Based on these findings, it is evident that identifying the spiritual needs of patients is crucial for nurses. The literature also supports this, indicating that recognizing and addressing the spiritual needs of patients facilitates acceptance of their condition, fosters inner peace, reduces anxiety, alleviates depression, and helps patients regain self-confidence and a positive self-concept [16, 31, 37, 38].

This study also has limitations. One important limitation is the lack of heterogeneity regarding the gender characteristics of both patients and caregivers, which may restrict the transferability of findings to male participants. Another limitation is the relatively small and single-center sample size, which may limit the generalizability of the results. Future research should aim to include larger and more diverse samples across gender, age, diagnosis, and cultural background, and should also employ longitudinal and interventional designs to explore how spiritual care interventions influence outcomes over time. By addressing these limitations, subsequent studies could provide more comprehensive insights into the spiritual needs of both patients and caregivers.

## Conclusion

This qualitative study explored the spiritual needs of patients receiving palliative care and their caregivers, shedding light on the challenges they encounter and the coping strategies they employ. The findings revealed that patients struggle with functional decline, psychological pain, and confronting death, while seeking acceptance, transcendence, and family support as key spiritual resources. Caregivers, in turn, face significant care burden and information deficits but draw spiritual strength from their devotion to God or a higher power and from the meaning they attribute to caregiving. Participants also reported receiving support from God, family, and close social networks during their most difficult times.

These results indicate that spirituality in palliative care is not only an individual matter but also a relational and family-centered phenomenon. At the same time, the coexistence of support resources with unmet informational and emotional needs underscores the necessity of approaching spiritual care from a multidimensional perspective. Clinically, the findings suggest that early recognition and systematic integration of spiritual care into practice can alleviate distress, strengthen resilience, and improve quality of life for both patients and caregivers. From an educational perspective, it is crucial to equip healthcare professionals with the skills to assess and address spiritual needs as part of holistic care. Furthermore, healthcare institutions should acknowledge the significance of spiritual care, develop structured training programs, and provide ongoing support mechanisms.

This study contributes to the scientific literature by addressing the spiritual needs of both patients and caregivers simultaneously, emphasizing that spiritual care is an essential component of palliative care. Future research should aim to include larger and more diverse samples, employ longitudinal designs, and evaluate the effectiveness of structured spiritual care interventions.

## Data Availability

Interview data generated and analyzed regarding palliative care patients and their caregivers during this study may be provided by the corresponding author with individuals anonymized due to ethical concerns and confidentiality agreements.
